# A Multi-Centre Study to Risk Stratify Colorectal Polyp Surveillance Patients Utilising Volatile Organic Compounds and Faecal Immunochemical Test

**DOI:** 10.3390/cancers14194951

**Published:** 2022-10-09

**Authors:** Subashini Chandrapalan, Farah Khasawneh, Baljit Singh, Stephen Lewis, James Turvill, Krishna Persaud, Ramesh P. Arasaradnam

**Affiliations:** 1 University Hospital of Coventry and Warwickshire, Coventry CV2 2DX, UK; 2 Warwick Medical School, University of Warwick, Coventry CV4 7AL, UK; 3 University Hospital of Leicester, Leicester LE3 9QP, UK; 4 University Hospital of Plymouth, Plymouth PL4 8AA, UK; 5 York and Scarborough Teaching Hospitals, York YO31 8HE, UK; 6 Department of Chemical Engineering and Analytical Science, University of Manchester, Manchester M13 9PL, UK; 7 Health, Biological & Experimental Sciences, University of Coventry, Coventry CV1 5FB, UK; 8 School of Health Sciences, University of Leicester, Leicester LE1 7RH, UK

**Keywords:** polyp surveillance, faecal immunochemical test, urinary volatile compounds, non-invasive tests, high-risk finding

## Abstract

**Simple Summary:**

A total of 15% of the annual colonoscopies are undertaken for polyp surveillance, and this causes strain on already stretched endoscopy services in the U.K. Hence, there is an urgent need to triage and prioritise patients with high-risk findings. The performance of the faecal immunochemical test is not adequate to be used within polyp surveillance. In light of this, we investigated the usefulness of urinary volatile organic compounds for the detection of high-risk findings. We confirmed that urinary volatile organic compounds can be used to triage patients with high-risk findings. It can be used alone or in combination with faecal immunochemical tests.

**Abstract:**

(1) Background: The service capacity for colonoscopy remains constrained, and while efforts are being made to recover elective services, polyp surveillance remains a challenge. (2) Methods: This is a multi-centre study recruiting patients already on polyp surveillance. Stool and urine samples were collected for the faecal immunochemical test (FIT) and volatile organic compounds (VOC) analysis, and all participants then underwent surveillance colonoscopy. (3) Results: The sensitivity and specificity of VOC for the detection of a high-risk finding ((≥2 premalignant polyps including ≥1 advanced polyp or ≥5 premalignant polyps) were 0.94 (95% CI, 0.88 to 0.98) and 0.69 (95% CI, 0.64 to 0.75) respectively. For FIT, the sensitivity was (≥10 µg of haemoglobin (Hb) / g faeces) 0.54 (95% CI, 0.43 to 0.65) and the specificity was 0.79 (95% CI, 0.73 to 0.84). The probability reduction for having a high-risk finding following both negative VOC and FIT will be 24% if both tests are applied sequentially. (4) Conclusion: The diagnostic performance of VOC is superior to FIT for the detection of a high-risk finding. The performance further improves when VOC is applied together with FIT sequentially (VOC first and then FIT). VOC alone or the combination of VOC and FIT can be used as a triage tool for patients awaiting colonoscopy within a polyp surveillance population, especially in resource-constrained healthcare systems.

## 1. Introduction

The prevalence of colonic adenomas ranges between 30% and 40% over the age of 60 years in an average-risk population [1,2]. The majority of colorectal cancer (CRC) develops from adenoma; however, 90% of colorectal adenomas do not progress to CRC [3]. Although the development of CRC from adenoma takes years to decades, individuals with polyp syndromes develop CRC earlier in their life (third to fifth decade). Regardless, polypectomy reduces CRC incidence and its related mortality [4,5,6].

So far, there have been no randomised trials examining the benefits of polyp surveillance for the reduction in CRC related mortality. A large observational study by the U.S. National Polyp Study Workgroup compared the CRC incidence in patients who underwent polyp surveillance, with patients who did not have their polyps removed, as well as those from the general-population registry (reference groups in this study) [7]. This study showed 70% to 90% reduction in the incidence of CRC in patients who underwent polyp resection during surveillance colonoscopies, in comparison to their reference groups. There have been several other studies that concurred with the above finding [8,9,10,11,12].

The current polyp surveillance programme in the U.K. is based on colonoscopy. Patients who are found to have polyps on their index colonoscopy, undergo further colonoscopy examinations, in a predetermined interval according to their risk category, recommended by the British Society of Gastroenterology (BSG) guidelines [13].

Colonoscopy-based surveillance was shown to reduce the incidence of CRC [14], and its sensitivity for the detection of advance adenoma ranges between 0.90 and 1.00. Although colonoscopy is the gold standard, it is an invasive procedure. Moreover, most of the patients who are undergoing polyp surveillance are 60 to 75 years of age and have multiple comorbidities. Patients undertake vigorous bowel cleansing, prior to their colonoscopy procedure, which can result in significant dehydration and electrolyte imbalance in those who are vulnerable. Colonoscopy also carries a small risk of serious complications, e.g., perforation and haemorrhage [15,16,17]. Colonoscopy-based surveillance programmes also have cost implications on national health systems [18]. On average, 350 colonoscopies are undertaken annually for polyp surveillance alone across hospitals serving catchments of 0.5 million people. Nationally, 15% of the 685,000 colonoscopies are performed for polyp surveillance [18]. The national tariff for an outpatient colonoscopy is £550 [19]. Hence, it is essential to seek an alternative surveillance modality, preferably a non-invasive one. 

Faecal immunochemical test (FIT) has been evaluated widely in this regard [20,21,22]. A large, prospective study showed FIT sensitivity of 56.6% at the threshold of 10 μg haemoglobin (Hb)/g faeces, for the detection of advanced adenoma (adenoma ≥10 mm) [23]. Although it was shown to reduce the colonoscopy related cost, FIT would miss 40% to 70% of advanced adenoma [23].

Our previous work showed FIT and volatile organic compounds (VOC) in combination could improve the CRC detection rates [24]. We therefore set to evaluate FIT and urinary VOC, on their own and in combination, as a potential non-invasive, risk stratification tool for patients undergoing polyp surveillance. The focus of this study was on patients who were already on polyp surveillance following previously detected polyps.

## 2. Methodology

### 2.1. Study Design and Participant Recruitment

This is a multi-centre prospective study involving secondary care sites in the U.K. The NHS trust organisations that participated were the University Hospital of Coventry and Warwickshire (UHCW), Leicester General Hospital, York and Scarborough Teaching Hospitals and the University Hospital of Plymouth. Although all four trusts were involved, the study was coordinated and led by UHCW. Ethical clearance was obtained from London-Bromley Research Ethics Committee, U.K. (19/LO/1614). This diagnostic study followed the Standards for Reporting of Diagnostic Accuracy (STARD) reporting guideline [25].

The inclusion criteria were (1) age 18 years or older, (2) no history of any cancer within the last 3 years, (3) no history of liver or renal impairment, and (4) not acutely unwell. Individuals who were on a polyp surveillance programme and due for colonoscopy between September 2019 and April 2021 were identified as potentially eligible participants. Patients who had gone through the multi-disciplinary team (MDT) for polyp resection were also identified through their respective MDT coordinators. The identified list of potential participants was then cross-checked against the inclusion criteria.

**Figure 1:**
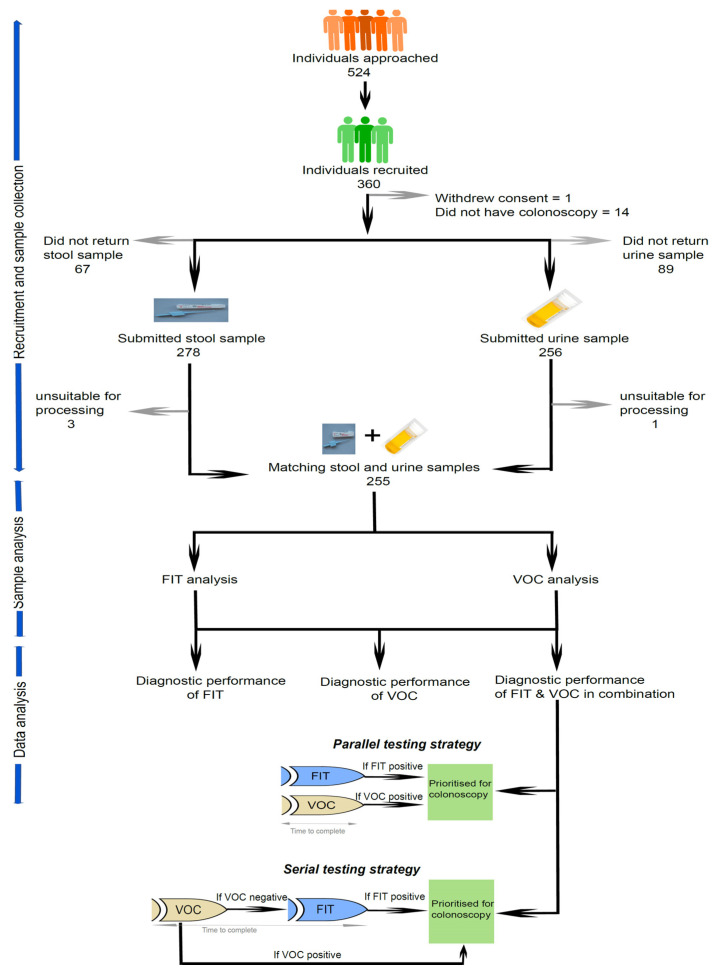
Participants flow through the study for the total number of individuals approached and recruited who met the inclusion criteria.

A pack containing an invitation letter together with a participant information sheet and consent form was sent out to these participants, prior to approaching them over the phone for consenting. Participants who consented were enrolled into the study. A second pack containing a pre-paid return envelope, FIT sample collection device together with a universal Sterilin sample pot for spot urine and pictorial instructions for sample collection was posted. Participants were then asked to collect stool and urine samples prior to taking bowel preparation. Figure 1 illustrates the participants flow through the study. As per the standard care practice, all of the participants then had a colonoscopy.

### 2.2. Stool Sample Collection and Analysis

A standard FIT picker stool retrieval kit for the HM-JACKarc FIT analyser (Kyowa Medex, Tokyo, Japan) was used to collect the stool sample. The stool sample was collected by participants within 4 weeks prior to having their colonoscopy. They were then posted to the regional Bowel Cancer Screening Hub (Rugby) in a pre-paid postage envelope.

Once contained within a FIT picker kit, the collected stool sample would remain stable for 14 days. Hence, for our study, samples that were older than 14 days from the time of collection were excluded from the analysis. A quantitative FIT analysis was undertaken using the automated HM-JACKarc analyser (Kyowa Medex, Tokyo, Japan), in accordance with the United Kingdom Accreditation Service, U.K. (UKAS) standard ISO 15189. The lowest limit of detection (LoD) for quantitative FIT was 3 µg Hb/g faeces and lowest limit of quantitation was (LoQ) 7 µg Hb /g faeces.

### 2.3. Urine Sample Collection 

Urine was specifically selected as a sample medium for the VOC analysis in this study, owing to its biological characteristics that favour its clinical utility. It undergoes a biological ‘pre-concentration’ in the kidneys, where a chemical ‘noise reduction’ takes place and the key ‘VOC signature’ concentration is increased [26]. We have developed a collection protocol based on previous studies to prevent degradation [27]. 

Spot urine samples were collected in two universal Sterilin bottles (20 mL each) from each participant prior to the administration of bowel preparation. The participants were requested to collect the urine samples in the morning and handover to their GP centres before 10 am on the same day. These were then transported to the respective trusts via the routine GP courier service (at room temperature) on the same day. Alternatively, the participants were asked to collect the urine samples the day before their bowel preparation, store that in their own fridges and bring it with them when they attended their colonoscopy appointment. These samples were then stored at the NHS trusts, in a freezer, at −80 °C within 12 h of collection. 

### 2.4. Urine Sample Analysis through E-Nose Technology

The analysis was performed using a SensAm analyser (SensAm Ltd., UK) at the Department of Chemical Engineering and Analytical Science, University of Manchester. This instrument was used in a number of studies previously and has been shown to be robust [28,29]. Moreover, its easy-to-use sample collection system and analysis allow bedside clinical application. 

**Figure 2:**
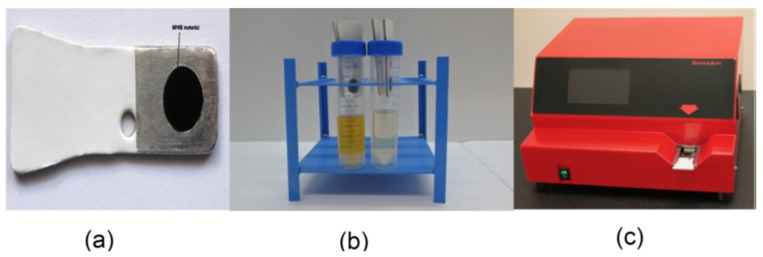
(**a**) Polymer tab used for solid phase microextraction. (**b**) Sampling urine headspace using these tabs (**c**) SensAm analyser. Images are published with the permission of SensAm Ltd., U.K.

In the laboratory, the frozen urine samples were thawed in a water bath at 30 °C for one hour. Then, the samples were transferred into the SensAm sampling system. This comprises adsorbent polymer-coated tabs, which have various levels of selective absorptive capacity for different volatile compounds. The tabs, once exposed, can be stored at room temperature for at least a week without a substantial loss of trapped volatiles and indefinitely at −80 °C. As such, two tabs (with hydrophobic and hydrophilic properties) were inserted into each of the urine sample headspaces through a slit-like opening on the lids of the sample bottles. (Figure 2a,b). They were left for 5 min to capture the VOC and reach chemical equilibrium. Thereafter, the tabs were removed and sealed in plastic receptacles. These tabs were then inserted into the SensAm analyser and heated up to 120 °C for two minutes to release the chemical compounds entrapped (Figure 2c). The SensAm analyser has an array of metal oxide sensors that were connected to a thermal desorption system. Five-point measurements between 20 s intervals were then taken from each Table. The instrument provides a multivariate response that is analysed to produce a fingerprint of the complex mixture of volatiles. This is processed using neural network-based pattern recognition [28]. The neural network is initially trained to differentiate between one pattern over the other. Thus, it can distinguish an abnormal pattern from a normal one and also the likelihood of having the disease for that particular pattern.

### 2.5. Identification of Chemical Compounds

A preliminary study was performed to identify the specific chemical compounds present (if any) in patients with polyps, using 6 samples (cases and controls). The gas chromatography and mass spectrogram (GC/MS) analysis was performed at the U.K. National Measurement Laboratory. Each sample was processed in two ways: methylation and salting out and salt out only. For GC (Agilent 7890A, Agilent Technologies, CA, USA), each sample tube was heated at 220 °C for 20 min. Desorbed VOCs were then transferred at 220 °C in 3:1 split injection ratio by the chromatograph injector using helium (as a carrier gas). Separation and quantification of the desorbed VOCs was performed by coupling the chromatograph with a mass spectrometer (Agilent 5975C), using a capillary column of 30 m × 0.25 mm internal diameter with a 1.4-μm film thickness (Agilent ZB-624) and at 35 °C oven temperature for 4 min. It is then ramped up to 250 °C by 10 °C per min until it reaches 250 °C. This heating was maintained for a further 5 min.

### 2.6. Colonoscopy Examination

All of the participants had undergone an endoscopic colonic examination as standard care. The endoscopy was performed by a Joint Advisory Group on Gastrointestinal Endoscopy (JAG) accredited endoscopist. In line with the JAG criteria, a colonoscopy was considered a complete examination if caecal intubation was achieved and confirmed by ileal intubation, identification of ileo caecal valve or the appendicular orifice.

### 2.7. Data Collection and Management

Once the participants were recruited in the study, a thorough medical history was obtained during telephone consultations. The details of the medical history, as well as the medications, were also taken. The participants’ general practitioners’ records were also cross-checked for accuracy.

Following colonoscopy examination, the electronic reports were reviewed for the following information: number of polyps identified, site of the polyps, size of the polyps, the endoscopic appearance of the polyps, whether the polyps were removed, and any other pathologies identified (inflammatory bowel disease, diverticulosis, microscopic colitis, etc.).

The histological diagnosis was obtained from the histology reports once they were available on the hospital’s electronic database. Participants were considered as having a high-risk finding if they had ≥2 premalignant polyps, including ≥1 advanced polyp or ≥5 premalignant polyps, in accordance with the BSG guidelines [13].

### 2.8. Statistical Analysis

#### 2.8.1. Sample Size Calculation

The sample size of the study was calculated to power the final outcome. The prevalence of adenoma in the polyp surveillance population was considered as 40% [13]. A previous study by Widlak et al. [24] showed that the sensitivity of FIT for the detection of adenoma was 0.53. The specificity was 0.68. If the study were to achieve 0.85 sensitivity, the upper limit of specificity would have been 0.5. Allowing for a margin of error of 0.7, the number of samples required to be analysed was 254. However, accounting for a dropout rate of 30%, the number of participants deemed necessary for recruitment was 350.

#### 2.8.2. FIT

The R software (The R Project for Statistical Computing, version 4.2.1) was used for all of the statistical analysis [30]. Participants with high-risk findings were compared with the rest of the study population (included participants with entirely normal colon, polyps that did not qualify under the high-risk category and other pathologies). The sensitivity, specificity, positive predictive value (PPV) and negative predictive value (NPV) of FIT were assessed at various cut-off levels. The rationales for the selection of different thresholds were: 3 µg Hb/g faeces—the lowest detection limit for the HM-JACKarc analyser; 7 µg Hb/g faeces—the lowest quantitation for HM-JACKarc analyser; 10 µg Hb/g faeces—it is the cut-off used in the urgent cancer referral pathway for symptomatic patients; 80 µg Hb/g faeces—it is the cut-off used in the Scottish bowel cancer screening programme; 120 µg Hb/g faeces—it is the cut-off used in the English bowel cancer screening programme; 150 µg Hb/g faeces—it is the cut-off used in the Wales bowel cancer screening programme; 5 µg Hb/g faeces, 20 µg Hb/g faeces and 100 µg Hb/g faeces—for explorative purposes. A receiver operator curve (ROC) analysis was performed to calculate the area under curve (AUC).

#### 2.8.3. VOC

**Figure 3:**
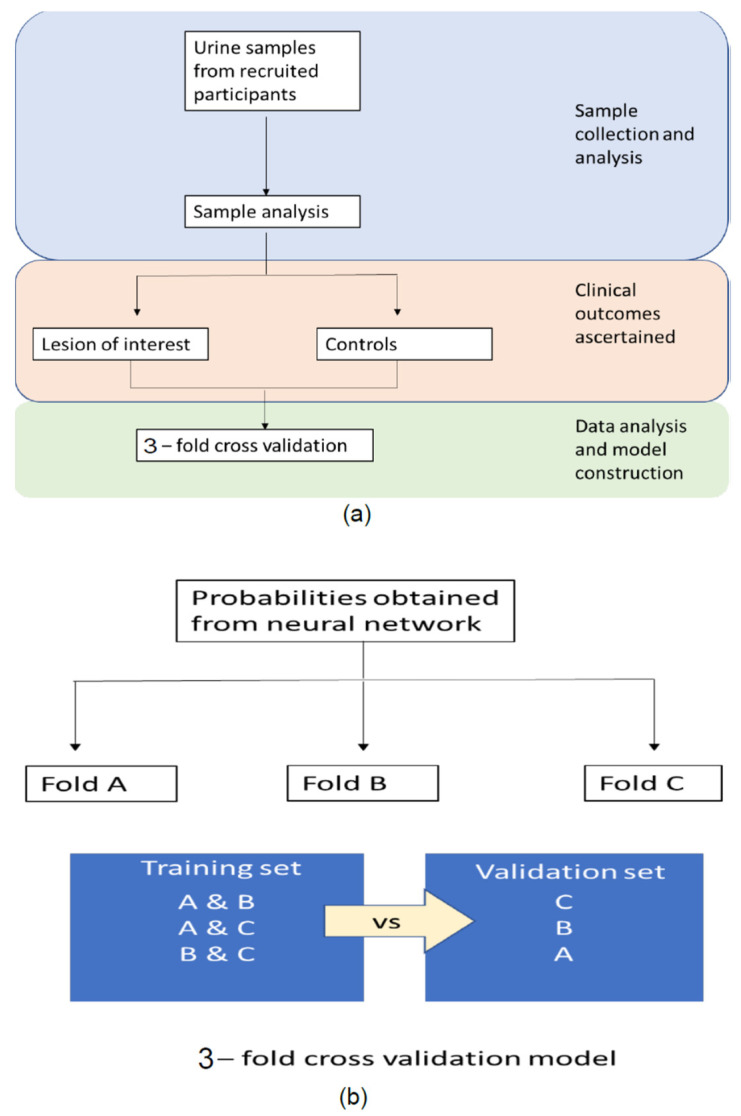
A schematic diagram to illustrate the k-fold cross-validation technique adopted in VOC data synthesis (**a**) steps in model construction (**b**) 3-fold cross-validation model.

The k-fold cross-validation technique was adopted for VOC analysis. Cross-validation is a sampling method used to evaluate machine learning models in a limited data sample, i.e., i.e., assessing the model performance in an unseen data set. See Figure 3. For our study, k was configured as 3 in view of the sample size. As such, the samples were randomised into three groups; these groups were called Fold A, Fold B, and Fold C. The first two folds were used for training the neural network, and the remaining fold was used for testing. This process was repeated until all three combinations of two folds were tested. The ROC curve was generated to calculate the AUC. The optimum threshold was determined using the ROC curve and by calculating the probability of participants having a high-risk finding in the context of the VOC measurements. Sensitivity and specificity at different threshold levels (two below and two above the optimum threshold) were evaluated.

#### 2.8.4. VOC and FIT Combined

As a second step, different testing scenarios were explored for the utilisation of FIT and VOC in combination: parallel testing and serial testing. In a parallel setting, FIT and VOC tests are given together, and the clinical condition is considered as not present if both tests are negative. For this, FIT values were fed into the neural network to combine with the VOC results in order to produce a single measurement. The neural network was retrained with and without FIT data using a complex mathematical vector method [31,32]. 

In serial testing, VOC and FIT are applied in a sequential manner, depending on the results of the first test. In our study, we considered VOC as the first test and FIT as the second test (FIT is given to patients who are negative for VOC). The pre-test and post-test probabilities of VOC and FIT were calculated using Fagan’s nomogram [33,34]. Fagan’s nomogram is a graphical tool for estimating how much the result of a diagnostic test changes the probability that a patient has a disease. This considers the prevalence of the disease and the precision estimates of the diagnostic test in question. 

A comparison table was also produced to illustrate the number of colonoscopies required, lesions detected, and lesions missed for colonoscopy-based surveillance vs. serial testing utilising VOC then FIT vs. parallel testing.

### 2.9. Patient and Public Involvement

Patient and public representatives were involved in the planning of the study through the Patient and Public Research Advisory Group at UHCW NHS Trust. The study design and all participant-facing documents (participant information sheet, consent form and sample collection information sheet) were presented for review and suggestions were incorporated for improved clarity.

## 3. Results

A total of 524 participants were approached for the study, and 360 were recruited. Amongst them, 255 participants provided matching urine and faeces samples that were suitable for analysis (Figure 1). The baseline patient characteristics are given in Table 1. 

### 3.1. Diagnostic Performance of FIT for the Detection of a High-Risk Finding (≥2 Premalignant Polyps including ≥1 Advanced Polyp or ≥5 Premalignant Polyps)

**Figure 4:**
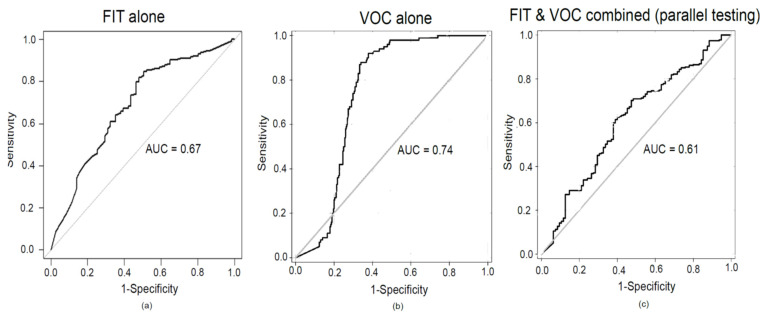
Receiver operator characteristics curve analysis for (**a**) FIT alone at 10 ug/g faeces, (**b**) VOC alone (**c**), FIT, and VOC in combination (when applied together) for the detection of a high-risk finding. FIT and VOC values were combined by neural networks through a complex mathematical vector method.

Table 2 illustrates the diagnostic performance at different thresholds. The sensitivity and specificity of FIT (at 10 µg Hb/g faeces), for the detection of a high-risk finding, were 0.54 (95% confidence interval (CI), 0.43 to 0.65) and 0.79 (95% CI, 0.73 to 0.84) respectively. The AUC for a high-risk finding was 0.67. See Figure 4a. The cut-off of 10 μg Hb/g faeces was specifically selected as the best trade-off between sensitivity and specificity. Relatively high sensitivity at this threshold is useful if the FIT is to be used as a rule-out test in the risk stratification of patients (a rule-out test for the possibility of having a high-risk finding).

### 3.2. The Diagnostic Performance of VOC for the Detection of a High-Risk Finding

The precision estimates for the detection of a high-risk finding (≥2 premalignant polyps including ≥1 advanced polyp or ≥5 premalignant polyps) at a threshold of 0.88 were: sensitivity of 0.94 (95% CI, 0.88 to 0.98) and specificity of 0.69 (95% CI, 0.64 to 0.75). The precision estimates at different threshold levels are given in Table 3. The ROC curve is shown in Figure 4b. The AUC was 0.74. 

### 3.3. The Combined Diagnostic Performance of VOC and FIT in the Detection of a High-Risk Finding

Here, we demonstrate how VOC and FIT could be utilised in combination within a surveillance setting. Fagan’s nomogram has been used as a graphical tool for this purpose. The sensitivity and specificity of FIT at the cut-off of 10 µg Hb/g faeces and the precision estimates of VOC at a threshold of 0.88 were considered for the scenarios below. 

#### 3.3.1. FIT and VOC in a Parallel Testing Manner

**Figure 5:**
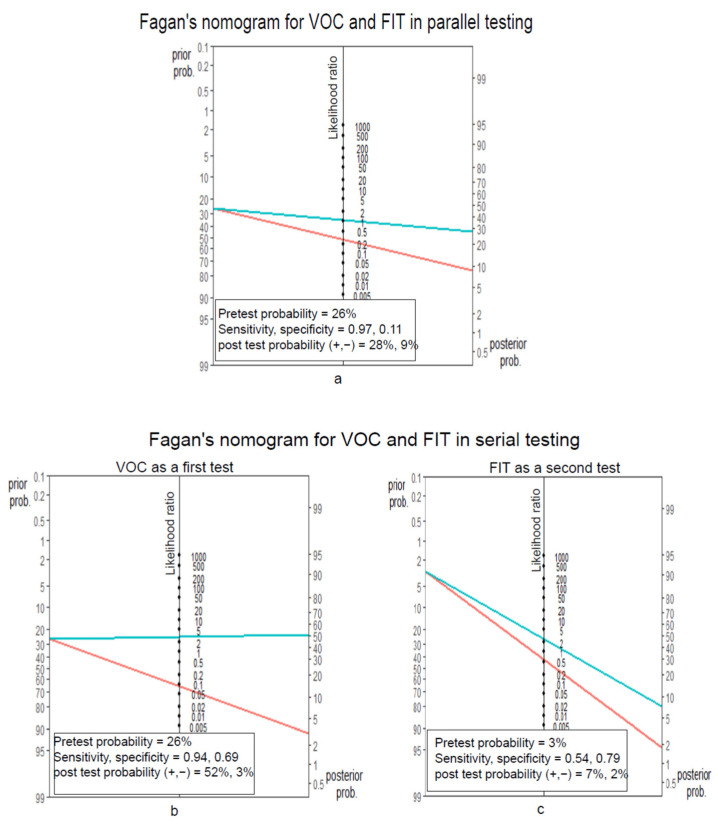
Fagan’s nomograms illustrating the pre-test and post-test probabilities of volatile organic compounds (VOC) and faecal immunochemical test (FIT) in (**a**) parallel testing and (**b,c**) serial testing, for the detection of a high-risk finding (as per the BSG guidelines. In serial testing, VOC is applied as a first test (**b**), and FIT is applied as a second test (**c**). The post-test probability of having a high-risk finding in a VOC-negative group has been considered as the pre-test probability for FIT. The probability reduction for parallel testing is 17%, and for serial testing, it is 24%. The green line corresponds to a positive test, and the red line to a negative test.

In this setting, FIT and VOC tests are given together, and the clinical condition is considered as not present if both tests are negative. The combined sensitivity was 0.97, and the specificity was 0.11. Adding the FIT data reduced the specificity of VOC. The AUC was 0.61. Figure 4c. Figure 5a Fagan’s nomogram shows the probability reduction of 17% for having a high-risk finding when both tests are negative. The post-test probability is 9%.

#### 3.3.2. VOC then FIT in a Serial Testing Manner 

VOC testing is used as the first surveillance test for calculations (sensitivity of VOC for the detection of a high-risk finding was higher compared to FIT; hence, VOC is used as a first test to maximise the initial patient capture). If a participant is negative for VOC, then the individual undergoes further testing with FIT. 

The prevalence of having a high-risk finding (high-risk finding is ≥2 premalignant polyps including ≥1 advanced polyp or ≥5 premalignant polyps) in the study was 26%. This is the pre-test probability for the VOC test. The post-test probability of having a high-risk finding after a positive VOC test would be 52%, and after a negative VOC test, it would be 3%. Now, the post-test probability of a negative VOC test would be the pre-test probability for FIT if a patient undergoes FIT testing following a negative VOC test. Thus, the post-test probability of having a high-risk finding following a negative VOC and FIT test would be 2%. The Fagan nomograms illustrating the above are presented in Figure 5b,c.

### 3.4. Comparison of Parallel and Serial Testing in 1000 Polyp Surveillance Patients 

Table 4 shows different testing scenarios and the number needed to scope for the detection of a high-risk finding. 

As per the current practice, if 1000 polyp surveillance patients undergo colonoscopy, the number of detected high-risk findings would be 260 (prevalence 26% in the study population); hence, the number of colonoscopies required to detect one high-risk finding would be 4. When VOC then FIT are to be used in a serial testing manner, all of the patients with a positive VOC undergo a colonoscopy and only those VOC-negative patients who are subsequently FIT-positive undergo a colonoscopy. Hence, the total number of colonoscopies required would be 590 (474 + 116) for 1000 patients. From the sensitivities of VOC and FIT, it can be calculated that the number with a high-risk finding detected would be 253 per 1000 patients (true positives for the tests) (see Supplementary File S1 for calculations). Thus, the number of colonoscopies performed to detect one high-risk finding would be two. By contrast, when parallel testing is considered, the number of colonoscopies needed to detect a high-risk finding would be 4. Therefore, utilising a serial testing strategy in polyp surveillance programmes may halve the number of colonoscopies needed to detect a single high-risk finding. However, it should be noted that 7 high-risk lesions would be missed per 1000 people tested (0.007%).

### 3.5. Volatile Organic Compounds Identified in Patients with High-Risk Findings

A total of 10 chemical compounds were predominantly found in patients with high-risk findings. These were (from the highest to lowest concentration) acetone, 2-Pentanone, toluene, 4-Heptanone, hexanoic acid, dimethyl trisulfide, o/p cymene, gamma terpinene, benzoic acid, and benzeneacetic acid.

## 4. Discussion

This is the first study that has assessed VOC and FIT in a pairwise manner within a polyp surveillance population. For the detection of a high-risk finding, VOC showed a better sensitivity profile, while FIT showed better specificity. This is likely due to the fact that FIT picks up bleeding polyps whereas VOC detects a change in polyp ‘microenvironment’. Therefore, FIT and VOC can be complementary for the detection of high-risk findings. The probability of having a high-risk finding following a negative VOC test would be 3%; administration of FIT following a negative VOC test would achieve a probability reduction of a further 1% if both tests are negative. It is noteworthy that different cut-off values could be applied when VOC and FIT are utilised in a sequential testing manner (as in this study). If VOC is used as a first test, then the FIT cut-off could be set higher to minimise false negatives.

**Figure 6:**
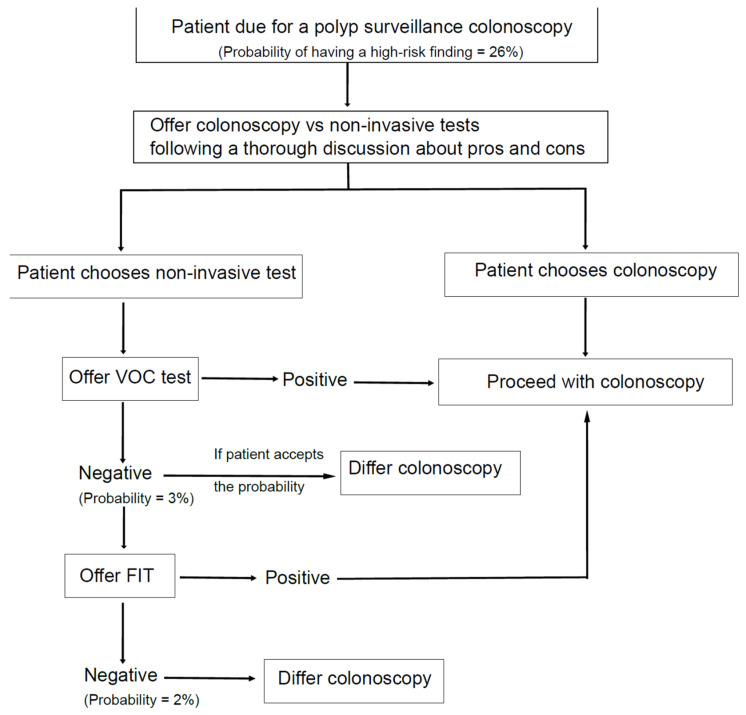
Proposed algorithm using non-invasive tests as a triage tool in polyp surveillance. Probability = probability of having a high-risk finding following a negative test.

As shown in our study, urinary VOC, either alone or in combination with FIT, has the potential to be utilised in polyp surveillance programmes, especially as a triage tool. Moreover, its easy applicability allows them to be sampled in a primary care setting. Considering the recent pandemic (year 2020/2021), the NHS healthcare system was under severe constraints and is still recovering from it. All routine procedures were postponed or delayed as the healthcare system went into an emergency mode. Maringe et al. [35] showed that the pandemic has resulted in a 15.3–16.6% increase in death rate; the number of referrals by GPs to hospital clinics for investigation of possible bowel cancer reduced by 63%, and the number of colonoscopies performed monthly for lower GI symptoms (suspicious of bowel cancer) also fell by 92%. Due to the new infection control measures, the endoscopy services are currently still functioning at 70% of their capacity. In such a scenario, non-invasive testing such as VOC and FIT could play a pivotal role in triaging patients for further investigations. Following a VOC or VOC and FIT, if a patient is deemed at increased risk of having a high-risk finding, then they could be prioritised for colonoscopy. See Figure 6. 

Stakeholders and patients might have differing views on adopting a non-invasive modality within polyp surveillance. This is largely dependent on cost, acceptability of missing lesions and patients’ preferences. Patients’ involvement in the decision-making process is very much encouraged. A recent national survey from the U.S. showed that most of the participants in CRC screening had preferred a collaborative decision process on whether to screen, the method to use and when to screen [36]. Patients who are on polyp surveillance usually undergo several colonoscopies at a predetermined interval. These groups of patients could make a choice of their preferred modality: colonoscopy vs. non-invasive tests. They could choose VOC only if the probability of having a high-risk finding (3%) is acceptable for them, or they could undergo further testing with FIT if they prefer a lower post-test probability. However, the cost implications of having FIT tests should be weighed against achieving a 1% of probability reduction for having a high-risk finding, especially if these tests are undertaken in population-based surveillance programmes. Further, an extensive discussion informing the detection rates, miss rates, advantages, and disadvantages of each modality (colonoscopy vs. non-invasive tests) should take place prior to such decision making. Of note, the cost for FIT and VOC is low (each £20), while the national tariff in the U.K. for colonoscopy is £550. 

It is interesting to note that none of the chemical compounds identified as unique to patients with high-risk findings on GC/MS. Previous studies showed that these compounds were also detected in patients with CRC [37,38]. While technical limitations may be contributory in part, it may also suggest that biologically, VOC profiles of those with high-risk findings are similar to those who harbour CRC. It is noteworthy that De Vietro et al. [39] in their study observed that both cancer cells and normal colonic mucosa (from the same patient) produced similar VOC chemical compounds but with differing fingerprints. 

### 4.1. Strengths and Limitations

Since it is a multicentered study, the findings from this study have suitable generalisability. Moreover, it is the first study to assess both biomarkers in a pairwise manner. When VOC and FIT are applied sequentially, different cut-off values may be used if VOC is applied first and FIT second. The effects of physiological factors such as age, sex and BMI on the performance of VOC were not evaluated in this study due to the small sample size. Similarly, single polyps that were greater than 10 mm and serrated lesions were not evaluated separately in this study (only 12 patients were found to have single 10 mm polyps and none with serrated polyps). Lifestyle factors, such as smoking and alcohol intake, could also have had an impact on the VOC chemical signature. The effects of drugs on the VOC profile have not been assessed in studies and hence remain unknown. However, our study reflects a real-life scenario, as adjusting for these factors would have practically been impossible if VOC were to be used in a mass surveillance setting. Family history was not evaluated in this study, which may have influenced the surveillance modality of choice. 

### 4.2. Future Research and Unanswered Questions

An ideal study should have multiple arms assessing all of the non-invasive options parallelly against the currently available gold standard with an opportunity to undertake subgroup analysis to further understand the mechanistic origin of VOC. 

## 5. Conclusions

In this study, we evaluated FIT and urinary VOC as non-invasive tools to risk stratify patients with high-risk findings. VOC is superior to FIT in detecting patients with high-risk findings. Although the combination of VOC then FIT performed better than any individual test alone, the cost implication of combining both tests in a population surveillance programme should be carefully evaluated. 

## Figures and Tables

**Table 1:** Demographics and baseline characteristics of the participants included in the analysis.

Characteristics	Value (n = 255)
Age, years	
Mean (standard deviation, SD)	67.65 (10.11)
Median (interquartile range, IQR)	69 (60.25 to 75.00)
Sex	
Female, n (%)	113 (44)
Male, n (%)	142 (55)
Body mass index	
Mean (SD)	27.81 (5.38)
Median (IQR)	27.32 (24.56 to 30.76)
FIT value, μg/g faeces	
Mean (SD)	44.91 (124.13)
Concurrent medication	
Proton pump inhibitors, n (%)	79 (30)
Anticoagulation therapy, n (%)	25 (10)
Antiplatelet therapy, n (%)	20 (8)
NSAID and aspirin, n (%)	27 (10)
Current alcohol consumption, n (%)	126 (49)
Smoking	
Current smoker, n (%)	23 (9)
Former smoker, n (%)	59 (23)
Never smoked, n (%)	173 (67)
Colonoscopy findings	
Normal, n (%)	73 (28)
All adenoma, n (%)	162 (63)
High-risk finding (among all adenoma), n (%)	68 (26)
Other pathologies (diverticulosis, microscopic colitis)	15 (6)
CRC, n (%)	5 (2)

**Table 2:** Diagnostic test accuracy of faecal immunochemical test for the detection of a high-risk finding. Prevalence = 0.26.

Cut-Off Levels	True Positives	True Negatives	Sensitivity	Specificity	PPV	NPV
At 3 ugHb/g faeces	42	123	0.62 (0.50, 0.73)	0.66 (0.59, 0.72)	0.39 (0.32, 0.45)	0.83 (0.77, 0.89)
At 5 ugHb/g faeces	38	136	0.56 (0.44, 0.68)	0.73 (0.66, 0.79)	0.42 (0.34, 0.49)	0.83 (0.78, 0.86)
At 7 ugHb/g faeces	37	140	0.54 (0.41, 0.65)	0.75 (0.69, 0.81)	0.43 (0.35, 0.51)	0.82 (0.78, 0.86)
At 10 ugHb/g faeces	37	148	0.54 (0.41, 0.65)	0.79 (0.73, 0.84)	0.47 (0.39, 0.56)	0.83 (0.79, 0.86)
At 20 μg Hb/g faeces	33	159	0.49 (0.37, 0.61)	0.85 (0.79, 0.89)	0.53 (0.42, 0.63)	0.83 (0.78, 0.85)
At 80 μg Hb/g faeces	17	170	0.25 (0.16, 0.37)	0.91 (0.86, 0.95)	0.50 (0.34, 0.64)	0.78 (0.74, 0.79)
At 100 μg Hb/g faeces	16	172	0.23 (0.13, 0.34)	0.92 (0.88, 0.95)	0.50 (0.35, 0.66)	0.77 (0.74, 0.89)
At 120 μg Hb/g faeces	14	172	0.20 (0.11, 0.31)	0.92 (0.88, 0.95)	0.47 (0.31, 0.63)	0.77 (0.74, 0.78)

**Table 3:** Diagnostic test accuracy of volatile organic compounds for the detection of a high-risk finding.

Cut-Off Levels	Sensitivity	Specificity	PPV	NPV
0.78	0.99 (0.94, 0.99)	0.48 (0.42, 0.54)	0.66	0.98
0.81	0.98 (0.92, 0.99)	0.54 (0.48, 0.60)	0.68	0.96
0.88	0.94 (0.88, 0.98)	0.69 (0.64, 0.75)	0.75	0.92
0.94	0.66 (0.55, 0.75)	0.76 (0.70, 0.80)	0.73	0.69
0.95	0.43 (0.33, 0.53)	0.77 (0.72, 0.82)	0.66	0.57

**Table 4:** Comparison of parallel and serial testing scenarios per 1000 polyp surveillance patients. Prevalence = 26% = 260/1000.

	Sensitivity	Specificity	Test Positives	Test Negatives	High-Risk Findings Detected among Those Tested Positive	High-Risk Findings Detected among Those Tested Negative	Total Colonoscopies Needed	Number Needed to Scope to Detect One High-Risk Finding
Colonoscopy for all of the patients on polyp surveillance programme (current practice)	N/A	N/A	N/A	N/A	260	N/A	1000	4
Parallel testing	0.97	0.11	911	89	252	8	911	4
Serial testing								
VOC as a first test	0.94	0.69	474	526	244	16	474	2
FIT as a second test	0.54	0.79	116	410	9	7	116	

## Data Availability

The data presented in this study are available on request from the corresponding author.

## Supplementary Materials

The following can be found at: https://www.mdpi.com/article/10.3390/cancers14194951/s1. File S1: Calculation of true positives and true negatives and 2 × 2 cross-tabulation for serial testing strategy.
