# Supplementary material for: A Multi-Centre Study to Risk Stratify Colorectal Polyp Surveillance Patients Utilising Volatile Organic Compounds and Faecal Immunochemical Test
Source: Cancers (Basel). 2022 Oct 9;14(19):4951. doi: 10.3390/cancers14194951 (PMC9562257; doi:10.3390/cancers14194951)
Supplement: Supplementary file 1 [file cancers-14-04951-s001.zip › cancers-1916094-supplementary.pdf]

In serial testing

2x2 table for VOC

	Disease +	Disease -	Total
VOC +	244	230	474
VOC -	16	510	526
Total	260	740	1000

2x2 table for FIT

	Disease +	Disease -	Total
FIT +	9	107	116
FIT -	7	403	410
Total	16	510	526

Prevalence = 26%

Number of patients with a high-risk finding per 1000 = 260

Therefore, patients without a high-risk finding = 1000-260 = 740

Sensitivity = TP/Disease positive

For VOC

0.92 = TP/260 = 239

0.62 = TN / 740 = 459

For FIT

0.54 = TP / 16 = 9
